# Estimating Occupancy Levels in Enclosed Spaces Using Environmental Variables: A Fitness Gym and Living Room as Evaluation Scenarios

**DOI:** 10.3390/s20226579

**Published:** 2020-11-18

**Authors:** Andree Vela, Joanna Alvarado-Uribe, Manuel Davila, Neil Hernandez-Gress, Hector G. Ceballos

**Affiliations:** 1 School of Engineering and Science, Tecnologico de Monterrey, Monterrey 64849, Mexico; a00829703@itesm.mx (A.V.); joanna.alvarado@tec.mx (J.A.-U.); ngress@tec.mx (N.H.-G.); 2 Big Data Enterprise and Artificial Intelligence Laboratory, University of the West of England Bristol, Bristol BS16 1QY, UK; manuel.daviladelgado@uwe.ac.uk

**Keywords:** occupancy estimation, environmental variables, Internet of Things, enclosed spaces, Machine Learning, energy efficiency

## Abstract

The understanding of occupancy patterns has been identified as a key contributor to achieve improvements in energy efficiency in buildings since occupancy information can benefit different systems, such as HVAC (Heating, Ventilation, and Air Conditioners), lighting, security, and emergency. This has meant that in the past decade, researchers have focused on improving the precision of occupancy estimation in enclosed spaces. Although several works have been done, one of the less addressed issues, regarding occupancy research, has been the availability of data for contrasting experimental results. Therefore, the main contributions of this work are: (1) the generation of two robust datasets gathered in enclosed spaces (a fitness gym and a living room) labeled with occupancy levels, and (2) the evaluation of three Machine Learning algorithms using different temporal resolutions. The results show that the prediction of 3–4 occupancy levels using the temperature, humidity, and pressure values provides an accuracy of at least 97%.

## 1. Introduction

The understanding and modeling of occupancy patterns have been identified as key contributors to achieve improvements in energy efficiency [1,2]. Therefore, the International Energy Agency (IEA), in their Annex 66 [3] and Annex 79 [4], highlights the need for further explorations in this area. For example, the prediction of occupancy status in buildings and homes can lead to energy savings, between 30% and 50% of total consumption [2,5]. It is estimated that buildings represent from 30% to 40% of the total consumed energy and that such energy came mainly from fossil fuels. Thus, it is expected that in the next 20 years, the total energy consumption for buildings will increase by up to 40% [5,6,7].

Several systems are benefited by the occupancy information gathered in buildings, mainly the Heating, Ventilation, and Air Conditioners (HVAC) system. Similarly, real-time occupancy information can enable and benefit other building management services, such as security, emergency systems, fire systems, flow management, and automated energy management [5,8,9,10]. However, occupancy detection systems face several challenges, such as reliability, adaptability, and accuracy.

The efforts to estimate occupancy in buildings can be classified into two categories: direct methods and indirect methods [8]. On the one hand, direct methods are based on technologies that can directly report the presence of human beings. Some examples of these technologies are video cameras incorporating people counting methods and infrared illuminators, Radio Frequency Identification (RFID) sensors, optical tripwires, Wireless Fidelity (WiFi), and Bluetooth Low Energy (BLE). Although direct methods have proven to be a viable mechanism for measuring occupancy, they present serious disadvantages, such as expensive hardware, privacy issues, intrusiveness, complex processing, and installation feasibility [1,5,8,10,11]. On the other hand, indirect methods derive occupancy information by measuring the effects of human beings in an enclosed environment, these effects can be changes in CO${}_{2}$ concentration, temperature, humidity, luminosity, sound levels, among others. To measure these environmental parameters, indirect solutions use the following technologies: microphones, vibration detectors, CO${}_{2}$ concentration monitors, as well as light, humidity, and temperature sensors. Indirect methods are considered to be a valid alternative to deal with the limitations of direct methods [8,11,12].

Another important topic regarding occupancy detection is the Internet of Things (IoT). IoT has increased drastically the number of devices connected to Internet and the computational power provided by them. Furthermore, one characteristic of the IoT devices is their significantly lower cost compared to equipment used in surveillance solutions or in existing commercial systems for occupancy detection [5,9,10]. This and other characteristics have led to the fast development of IoT, which in turn has driven Edge Computing (EC) [10].

These benefits have sparked the interest of researchers on these technologies. For example, Parise et al. [13] developed an open-source IoT architecture for occupancy detection based on temperature, humidity, and pressure as environmental parameters, and composed of an EC environment and a cloud-based infrastructure. Similarly, Zemouri et al. [10] used EC devices and a cloud-based platform to detect occupancy in office buildings. In the same way, Adeogun et al. [12] detected and estimated occupancy in an office building by implementing an IoT LoRa (low-power wide-area network protocol) monitoring system based on a combination of temperature, humidity, light, and CO${}_{2}$ (as environment parameters), nine different sensors, and more than 20 nodes.

Nevertheless, despite many efforts by researchers to solve the problem of detecting occupancy, little attention has been paid to building a standard dataset that allows different algorithms to be easily compared. For the specific case of indirect techniques for occupancy detection, there are only a few publicly accessible datasets [14,15,16,17] but most of them are poorly documented or have not been used in formal research. There are two datasets that can be used for occupancy detection: Occupancy Detection Dataset [18] and Ecobee’s Donate Your Data program [19]. Occupancy Detection Dataset is from the University of California, Irvine (UCI), which contains seven features and more than 20,000 instances [18]. While this dataset is publicly available, it lacks information related to the number of people or the occupation range, which represent important attributes to estimate the occupation. Ecobee’s Donate Your Data program is made up of a large amount of data from various residences at different seasons of the year [19]. However, this dataset does not have the ground truth regarding occupancy and the access is not guaranteed (it can only be accessed upon request from Ecobee).

Regarding the occupancy estimation, several works have been carried out based on one or more environmental variables [1,8,11,12,20,21,22]. However, few works that did not use non-environmental variables as additional support for the estimation were found. On the one hand, there are works, such as Adeogun et al. [12], which achieved an estimation accuracy of 0.91 using pressure, humidity, and CO${}_{2}$, in addition to a Passive Infrared (PIR) sensor. A second example is the work of Chitu et al. [21], which in addition to using CO${}_{2}$, also registers the state of all the airflow sources, obtaining an accuracy of 0.69. On the other hand, among the works that only use environmental variables, Jiang et al. [22] and Zhou et al. [11] were found. Both used CO${}_{2}$ to estimate occupancy, obtaining an accuracy of 0.77 and 0.82, respectively. Another example is the work of Viani et al [8], which obtained an accuracy of 0.82 using temperature, humidity, and CO${}_{2}$. This is another of the few works in which temperature and humidity are considered to estimate occupancy. It is relevant to mention that there are no research works based only on environmental variables that do not use CO${}_{2}$. Furthermore, none of the studies based only on environmental variables presents an accuracy similar to that obtained by works using environmental and non-environmental variables, such as Adeogun et al [12].

Thus, this research work aims to generate an open dataset consisting of environmental variables collected from real scenarios (indirect method) as well as evaluating different Machine Learning algorithms on this dataset in order to estimate the occupancy level in an enclosed space. Thereby, the main contribution of this article is two-fold:

1. Two datasets built with real-world information: one dataset collected from a fitness gym and another gathered from the living room in a house inhabited by seven people.
2. The evaluation of three Machine Learning algorithms: Support Vector Machine (SVM), k-Nearest Neighbor (kNN), and Decision Trees (DT), to estimate 3-4 occupancy levels with different temporal resolutions using the built datasets and only the temperature, humidity, and pressure values.

It is important to point out this work is focused on estimating occupancy based solely on internal environmental conditions in enclosed spaces; external environmental conditions are out of the scope of this research. Furthermore, the internal environment is considered static, as temporal dependency in the data is not taken into account. In addition, even though the proposed solution design took into consideration integrating with other building management services, the real integration with such systems is out of the scope of this research as well.

The rest of this document is organized as follows. Section 2 provides the related work to the detection and estimation of the occupancy in enclosed spaces. Then, Section 3 addresses the methodology carried out in this approach and the approach itself. Subsequently, Section 4 presents the results of the proposed experimentation as well as its discussion. Finally, Section 5 gives the conclusions and future work.

## 2. Related Work

This work focuses on estimating occupancy in enclosed spaces using Machine Learning algorithms and environmental variables. To later use these results to reduce the energy consumption in buildings derived from HVAC systems, which are powered mainly by fossil fuels. In this section, relevant literature for this research is reviewed.

Of the multiple environmental variables that exist to determine the presence of human beings, temperature, humidity, and CO${}_{2}$ are among the most frequently used [1,5,7,8,9,10,11,12,13,18,20,21,22]. A reason for this is the large variety of sensors available on the market and their accessible cost when compared to more traditional solutions, such as weather stations. Among the works that use these variables, three approaches were found: (1) those based on temperature and humidity, (2) those based on CO${}_{2}$, and (3) those based on a model that combines the three mentioned variables plus others.

Among the most recent works based on temperature and humidity, the research of Zemouri et al. [10] and Huchuck et al. [7] were found, which proposed solutions to detect occupation based mainly on these variables and excluding the use of CO${}_{2}$. They obtained an accuracy of 0.83 and 0.75, respectively, despite that their research works were limited only to the detection problem; that is, the authors did not carry out any estimation approach, which is generally considered to be a more difficult task. Moreover, both approaches obtained lower accuracies compared to other approaches [5,13]. On the other hand, among the works focused on CO${}_{2}$, the studies of Jiang et al. [22] and Chitu et al. [21] were identified, which went one step further and tackled the problem of estimating the level of occupation in ranges (high, medium, and low). They obtained an accuracy of 0.77 and 0.69, respectively. As mentioned before, for the estimation issue the accuracy is a little lower than in the detection, however, there are works with multiple use of environmental variables that have obtained a significantly higher accuracy (0.91) [12].

An effective way to obtain better results is by using multiple environmental variables together, specifically, temperature, humidity, and CO${}_{2}$ [21]. This was the approach followed by Parise et al. [13], obtaining a detection accuracy of 0.96. Another example is the work of Kumar et al. [5], which obtained also for detection accuracy of 0.99. Another way in which researchers have tried to increase accuracy is by adding the state of doors and windows to the set of features used by the algorithms. Cali et al. [20] explored this idea, finding that with the 4.9% of the time that a window is open it is enough to have benefits in the accuracy of the model. Adeogun et al. [12] also incorporated this idea into their work. They trained a detection and estimation model using the temperature, humidity, CO${}_{2}$, windows, and doors; and used a two-layer Feedforward Neural Network (FNN), obtaining an accuracy of 0.94 for detection and 0.91 for estimation.

Although very good levels of accuracy have been achieved for detection and estimation, little has been done to achieve a generalized solution that can replicate these results in different spaces. This gap is addressed by Adeogun et al. [12], where they tried to use a trained algorithm in one room to perform the prediction in another room, obtaining a significant reduction in accuracy, from 0.94 to 0.60. Another important factor to consider is that few works have used only the three environmental variables mentioned above without the support of other auxiliary variables or sensors. For example, the work of Kumar et al. [5], which used the UCI dataset [18], used also light measurement which, for the case of detection, has been shown to be one of the variables that best predicts occupancy for the interior of the locations [12]. One of the most outstanding works is the work of Viani et al. [8], which only uses temperature, humidity, and CO${}_{2}$ without the help of supporting variables. They used only these three variables and along with a network of 29 IoT sensors, trained an SVM to estimate the occupation, obtaining a promising result of 0.82 of accuracy. Using only these three variables is a very important matter because unlike other variables, it is common for HVAC systems in buildings to already have such information available; and in the absence of this, the necessary sensors can be installed in a simple, non-intrusive, and low-cost way [22].

Another problem that can be noticed among the different works is the difficulty in comparing results due to the difference in the scale of the demos implemented for the experimentation of each one. These variations occur in both the temporal resolution and the spatial resolution of the experiment. For example, in the case of temporal resolution, the range of the experiments varied from eight days [20] to a full year [7], and in the case of spatial resolution, the scale is extended from one room [13] to an entire building [8]. An additional factor that could be added is the number of devices used, where a variation from one [10] to 29 devices [8] was found. Likewise, there are significant variations in the number of people involved, from three [10] to 28 people [22]. It is important to note that as the size of the demo increases (mainly in spatial resolution and the number of people in the case of estimation), a reduction in the maximum accuracy achieved is perceived. For example, the works [11,12,13] which achieved high accuracy results in the case of estimation used four to six people and no more than two rooms in their experiments.

Regarding the algorithms used for the detection and estimation of the occupation, three categories were found: (1) Physical Models, (2) Machine Learning, and (3) Deep Learning. In the first category, the mass balance equation is found and used almost exclusively with works based on CO${}_{2}$ concentration. Cali et al. [20] used this technique to estimate the occupation of a room for eight days, obtaining a correct estimation in 79% of the monitored period. Lately, this approach has lost interest because, in general, better results have been achieved with techniques of the other two groups [5,12]. As a consequence, currently, Machine Learning and Deep Learning are the most explored approaches among researchers. In the Machine Learning category, some of the most frequent algorithms are SVM, kNN, Random Forest (RF), Hidden Markov Model (HMM), Extreme Learning Machine (ELM), and Logistic Regression (LR) [1,5,7,8,9,10,13,21,22]. Regarding the Deep Learning category, algorithms like Long Short-Term Memory (LSTM), FNN, and Deep Forest were identified [7,11,12].

A final factor to note is that the vast majority of the articles reviewed used a dataset of their own elaboration, and only three of them worked with a dataset provided by other researchers [5,7,21]. This is important because it represents one more obstacle to make a fair and objective comparison of the results obtained in each work. Likewise, it was found that some of the datasets that were used in previous scientific publications are not publicly available to the community for further experimentation. This is also a major drawback to the development of the occupation estimation field. Table 1 presents a condensed summary of the reviewed literature.

As a conclusion for this review of related work, it was found that good levels of accuracy have been reached for both detection and estimation of occupancy. However, variations in the spatial and temporal scale, number of sensors, and number of participants make it difficult to accurately compare the different obtained results. Likewise, it was found that there are few works based solely on the use of environmental variables, such as temperature, humidity, and CO${}_{2}$ without the help of other variables or sensors (Light and PIR mainly), indicating that this approach needs to be explored. It is also noticed the existence of a trend towards preferring Machine Learning and Deep Learning techniques over physical models. Therefore, this work aims to focus on the gaps in the field of occupation mentioned above, developing a Machine Learning model based only on the use of environmental variables, which allows obtaining better results than those in the current literature.

## 3. Estimating Occupancy Levels in Enclosed Spaces Using Environmental Variables

Firstly, the methodology followed in this approach is presented. Subsequently, the research and development of the proposed estimation approach are described.

### 3.1. Methodology

This research involves collecting environmental data from enclosed spaces that will be addressed through Machine Learning techniques to predict occupancy levels in those spaces. Therefore, the methodology followed includes both activities related to the data collection, preprocessing, and exploration, as well as the selection and evaluation of the Machine Learning algorithms used to estimate occupancy levels. These activities are listed below.

- **Definition of the collection sensors.** The sensors used to collect the data are of utmost importance since the quality of the data available for further analysis and experimentation will depend on them. For the specific case of this research, sensors capable of collecting temperature, humidity, and pressure values with a resolution of 1 s are required in order to obtain a high-resolution dataset. This feature is key as one objective of this research is to explore the effects of data resolution on the prediction of occupation.
- **Data collection.** Two different locations will be selected for the data collection. This will allow obtaining independent datasets to contrast and enrich the results obtained from the experimentation.
- **Data preprocessing.** A curation of the data will be performed to remove missing values, analyze and update/remove sensor reading errors, standardize collected data, and integrate the labels corresponding to the occupancy levels for each record. All this processing will improve the quality of the collected data for further experimentation and publication.
- **Data exploration.** A visual exploration of the data will be carried out to find patterns and characteristics that could help in the interpretation of the results produced by the predictive models.
- **Selection of prediction models.** Three Machine Learning models widely used in the detection and estimation of occupancy levels will be selected through a review of the literature.
- **Design of experimentation and evaluation scenarios.** Experimentation scenarios will be designed to find an optimal subset of attributes as well as the optimal resolution of the dataset. In the same way, assessment scenarios will be proposed using the selected evaluation metric, which will allow comparing the results to determine the best estimation algorithm.

### 3.2. Definition of the Collection Sensors

**Figure 1:**
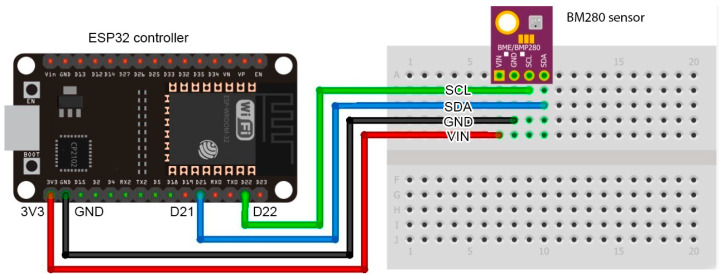
Electronic circuit used for data collection.

For data collection, an electronic circuit made up of an environmental sensor model BME280 [24] from Bosh and an controller model ESP32 [25] from Espressif Systems was designed, as shown in Figure 1.

On the one hand, BME280 is a low-cost, low-energy consumption sensor designed for meteorological monitoring. This sensor allows measuring the relative humidity (%), temperature (${}^{{}^{\circ}}$C), atmospheric pressure (hPa), and altitude (m) of the space in which it is located. Sensor ranges and accuracy are listed below: pressure 300 to 1100 hPa (±1 hPa accuracy), temperature −40 to 85 ${}^{{}^{\circ}}$C (±1 ${}^{{}^{\circ}}$C accuracy), relative humidity 0% to 100% (±3% accuracy), and altitude from 0 to 30,000 ft (±1 m accuracy) [24]. These characteristics make it suitable for the purposes of this research.

On the other hand, the ESP32 controller is a low-cost, low-energy consumption microcontroller with wireless connectivity, i.e., WiFi and Bluetooth. It supports the security standards WPA and WPA2, which makes it able to connect to modern wireless networks, and has a dual-core 32-bit 160 MHz processor and 520 KiB of static random access memory (SRAM). Additionally, ESP32 has software development kits for the main IoT platforms (e.g., Arduino), which facilitates its integration with other solutions. Hence, these characteristics make it suitable for a wide range of projects, including IoT [25].

### 3.3. Data Collection

The locations selected for the data collection are a fitness gym at a university and a private residential home. These locations were selected because of their contrasting characteristics in environmental and design-use conditions. On the one hand, the fitness gym has an AC unit and mechanical ventilation operating 24 h. It also has a capacity for 30–40 people to exercise aerobically and anaerobically. These high-intensity activities are expected to increase the occupants’ body temperature and exhalation rate, which could impact the enclosed space’s temperature and humidity saturation [26]. On the other hand, a living room is a significantly smaller space with a capacity for up to 10 or 12 people. It does not have mechanical ventilation, but it has an AC unit and a roof fan, which are turned on at specific times. This space is designed to relax and, in general, to do low-intensity activities. Thus, the contrasting characteristics of these locations help to ensure the proposed approach will be validated under various environmental conditions. Both locations are described below.

#### 3.3.1. Fitness Gym

The monitored space corresponds to the employees fitness gym of the Tecnologico de Monterrey in Mexico. Such a facility has an AC system with mechanical ventilation, which is an important factor due to AC systems try to minimize variations in temperature.

The collection was carried out over six days between 18 September 2019 and 2 October 2019. The data was measured in sessions of approximately 20 min at three different times of the day: morning, afternoon, and night. These schedules correspond to the periods of greatest traffic of people in the gymnasium reported by the gym manager, reason why these periods were chosen for data collection. The resolution of the measurements was one second, obtaining approximately 10 k records corresponding to about 180 min.

Due to the layout of the space and the limitations of available resources, it was not possible to use auxiliary cameras or sensors to record the ground truth. Thus, the data indicating the occupation range was entered manually by the person responsible for the collection. These occupation ranges were established and labeled in the following three levels: L (low), M (medium), and H (high). However, the number of people observed was not recorded.

#### 3.3.2. Living Room

The monitored space corresponds to the living room of a residential building. The monitored area is approximately 8 × 4 m and has mechanical ventilation: an AC unit, a ceiling fan, and sometimes, a floor fan. Furthermore, this space lacks windows and therefore has no natural ventilation.

**Figure 2:**
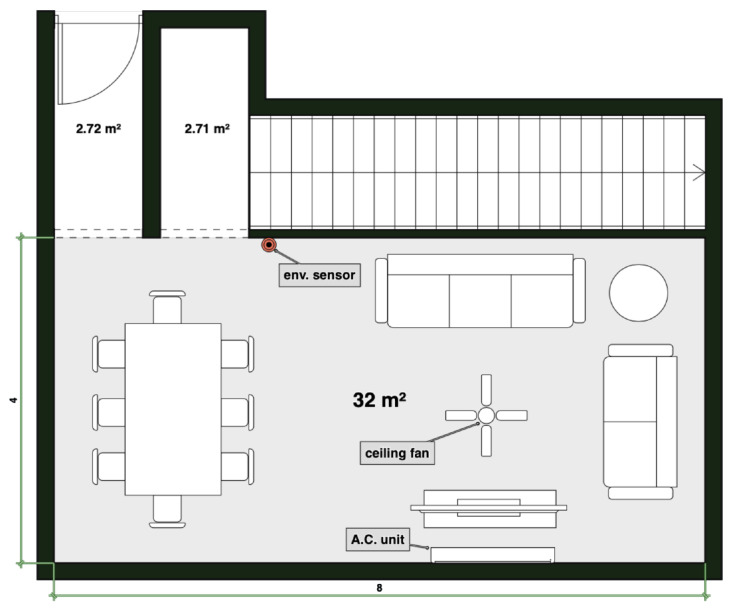
Sketch of the living room. The locations of the environmental sensor, ceiling fan, and AC unit are shown. The monitored area is approximately 32 m${}^{2}$.

The electronic circuit was carefully placed between the living room and the dining room, approximately five meters away from the AC unit. This in order to prevent the sensor from having direct contact with the air flow coming from the AC unit, if this was turned on. A sketch of the living room is shown in Figure 2.

The collection was carried out for 11 days between 14 May 2020 and 4 June 2020 in sessions with an average duration of 12 h, although some sessions lasted 24 h (there are periods of inactivity corresponding to early morning). The resolution of the measurements was one second, obtaining approximately 300 k records corresponding to 5000 min.

Regarding the ground truth, a camera was used to take photos of the living room every 10 min and therefore, the number of people can be recorded. This is an improvement over the fitness gym dataset as the exact occupancy is an unbiased value. Thereby, the maximum registered occupancy was seven people and the minimum was zero.

### 3.4. Data Preprocessing

To carry out the preprocessing of the collected data, the datasets were exported from the cloud storage as CSV files. In the case of the living room, each measurement day was stored in a different file and additionally, the ground truth of the entire collection was also recorded in a separate file. When crossing the files, small periods were found where there were data measurements but not their ground truth. This due to technical details during the collection, i.e., camera storage limitation. These periods were a minority, representing less than 1% of the data. As they were few records, it was decided to discard them.

Likewise, when the datasets corresponding to the fitness gym and living room were checked for missing values, only six measurements with missing values were found. Hence, these missing values were completed using the average value of the previous instance and the next instance. This solution was considered adequate since the high resolution of the dataset compared to previous works that used lower resolutions (1 min or more) [5,10,12,21].

Finally, the altitude information was discarded from the final datasets. This is because the altitude is not related to the problem of occupation, therefore, it makes no sense to leave such information. Moreover, according to the technical sheet of the BM280 sensor [24], the altitude information is calculated using pressure measurements, thus, the discriminant capacity that this variable could have would be contained in the pressure variable. Similarly, this relationship was verified by a Pearson correlation analysis in which both variables showed a perfect positive correlation; that is, their correlation was 1.0.

#### 3.4.1. Establishing the Occupancy Levels

As mentioned in Section 3.3, in the case of the fitness gym, the occupancy levels were recorded directly by the person responsible of the data collection. These levels are L (low), M (medium), and H (high). For this dataset, the level corresponding to Empty was not recorded. However, since the data collection was carried out during the busiest times of the fitness gym, it is unlikely that there was an empty period. As the number of people corresponding to each level had not been established and recorded, the values of the occupancy levels are subjective.

In the case of the living room, as mentioned above, the occupation records were obtained from photographs taken at ten-minute intervals. Thereby, the occupancy levels were established taking proportional intervals between the maximum and the minimum number of people observed, as described in [1]. Hence, since the maximum number of people recorded was seven, the occupancy levels were established as follows: Empty (0), Low (1–2), Medium (3–5), and High (6–7).

#### 3.4.2. Generating of Datasets with Different Resolutions

For each collection scenario, eight additional datasets were generated using the following resolutions: 10 s, 30 s, 1 min, and 5 min. That is, two datasets were generated for each resolution: the first one, taking only one sample for each period and the second one, averaging the samples of the selected resolution.

In the averaged datasets, for each feature (humidity, pressure, and temperature), two extra features were generated, which are kurtosis (kurt) and standard deviation (std), having a total of nine features for each dataset. The reason to add these features is that the information generated, related to the data for a given resolution window, can be useful to differentiate the occupancy levels. For instance, the std measures provide how close or far the values are to the mean, while kurt measures represent how frequent the extreme values can be expected. This idea is supported by the work of Zemouri et al. [10], where the standard deviation of environmental measurements (in conjunction with some de-trending techniques) were used to detect the occupancy with an accuracy of 0.87.

#### 3.4.3. Partitioning and Balancing

Each dataset was partitioned using 20% for testing and the rest for training. During this process, it was found that the datasets were imbalanced. This represents a problem because Machine Learning models are frequently becoming biased towards the majority class, hence, rendering them useless to identify the minority class. An effective way to mitigate this problem is by performing an oversampling to balance the classes represented in the datasets. For this purpose, the Adaptive Synthetic (ADASYN) algorithm [27] was used.

The basic functioning of ADASYN is as follows: First, the number of instances of the minority class to generate is calculated. Then, the “difficulty” of each instance $x_{i}$ belonging to the minority class is calculated, taking the kNN and calculating the ratio $r_{i}=\Delta_{i}/K$; where $\Delta_{i}$ is the number of neighbors belonging to the majority class for $x_{i}$ instance. Afterward, the ratio $r_{i}$ is used to calculate the number of instances $g_{i}$ to generate for each instance $x_{i}$ belonging to the minority class. Finally, the $g_{i}$ instances for each $x_{i}$ in the minority class are generated as follows: an $x_{zi}$ is randomly selected from the closest neighbors to $x_{i}$, and after that, the instance $s_{i}=x_{i}+\left(x_{zi}-x_{i}\right)*\lambda$ is generated; where λ is a random number ∈[0,1].

Two important differences are found among ADASYN and other balancing algorithms (e.g., SMOTE): (i) ADASYN focused on generating new instances using the most “difficult” instances, and (ii) ADASYN introduces a random error λ to reduce linear dependency between instances. These differences make it a preferred choice over SMOTE.

Therefore, after applying the ADASYN algorithm over the fitness gym and living room datasets, the size of the training datasets before and after balancing are shown in Table 2.

### 3.5. Data Exploration

For each scenario, the original dataset with the one-second resolution is used to perform the data exploration. First, variance analysis is carried out to understand how much measurements change between occupancy levels. Then, a time-line visualization of the environmental variables and a three-dimensional plot of the humidity, temperature, and pressure values is performed to identify any possible pattern.

### 3.6. Selection of Prediction Models

For the occupancy prediction, three classification models frequently used in the reviewed literature were selected [8,9,10,13]. Each model has different configuration parameters, which allow its performance to be adjusted. The fundamental operation of each model is described below:

- k-Nearest Neighbor (kNN): This is a classifier based on the concept of similarity. It finds the *k* neighbors most “similar” to the instance to be classified, and assign the statistical mode as the class of the new instance. Although there are several similarity measures, such as Manhattan, Minkowski, and Chebyshev, Euclidean distance was selected for this work because of its ease of being generalized in domains of *n* features [28]. Another important parameter is *k*, the number of neighbors used to classify each point. It is important to choose the value of *k* carefully since a low value increases the model bias but a value too high produce overfitting and an increment in the computational cost.
- Support Vector Machine (SVM): This algorithm can be used for classification and regression. It works by performing a projection of the data points to a higher dimension, where they can be separated using a line or a hyperplane [29]. The points that lie exactly at the limit of the decision surface are called Support Vectors. For the systemic search of the optimal hyperplane, a kernel function is used. Examples of some kernels are (but are not limited to): linear, polynomial, and radial base function (RBF). Besides the kernel, other important parameters of the model are the regularization parameter C and Gamma, which control the tolerance to classification errors and how large the area of influence of the decision boundary is (for non-linear kernels), respectively.
- Decision Trees (DT): This classifier works by creating a tree based on the features. Each non-leaf node of the tree represents a partitioning rule of the dataset using some attribute. The tree is recursively created by calculating the importance of each feature after a split. An important benefit of DT is that the result can be easily interpreted, i.e., to understand why a specific point was assigned to a class, just read the rule at each node. Among the parameters to optimize the model are the maximum depth, the minimum number of instances to make a separation, and the criteria to determine the importance of each attribute [28].

### 3.7. Design of Experimentation and Evaluation Scenarios

To test the prediction capabilities of the selected models, two experiments were designed. Each one of the experiments was applied to the fitness gym and living room datasets. In each case, an exhaustive search was made to find the best set of parameters using the training splits along with stratified cross-validation of five-folds and four repetitions. Thereby, the corresponding test split of each dataset was used to obtain the final scores on each experiment. The proposed experiments are described below:

- *Feature Selection*: To find the optimal subset of features, two feature selection techniques were implemented: (i) recursive feature elimination (RFE), and (ii) k-best with ANOVA F-value as score function. Chi-square was discarded as a score function due to the data contains negative values. This experiment was executed on the averaged 10-s datasets. This is because they are the highest resolution datasets that contain the extra attributes (kurtosis and standard deviation). For each scenario, the prediction was made using the following datasets:
  - FULL: 10 s averaged dataset with nine attributes (including kurtosis and standard deviation for humidity, pressure, and temperature).
  - RFE: 10 s averaged dataset with five best features selected by the RFE technique.
  - KBEST: 10 s averaged dataset with five best features selected by the k-best technique.
  - MIN: 10 s averaged dataset with three features (humidity, pressure, and temperature).
- *Resolution Selection*: Predictions using the distinct resolution datasets were performed: 10 s, 30 s, 1 min, and 5 min. Additionally, predictions were also made using the corresponding averaged datasets to contrast the performance of the models using a single sample against the averages.

#### Evaluation Metrics

Accuracy was used to evaluate the three selected models. As the prediction of the occupancy level is a multi-class problem, the macro-average was used to obtain the final value in each experiment. This is the approach followed by other works in occupancy detection [1,12]. The accuracy expresses the proximity of the obtained value to the real value. One of its benefits is that it takes into account the predicted negative values correctly. Formally the accuracy is defined as follows:

$$\begin{array}{c} Acc=\frac{TP+TN}{TP+TN+FP+FN} \end{array}$$

where *TP* corresponds to true positives, *TN* to true negatives, *FP* to false positives, and *FN* to false negatives.

## 4. Results and Discussion

Firstly, the data visualization carried out to explore the collected data is presented. Subsequently, the results and findings related to the evaluation scenarios for the fitness gym and living room datasets are described. Finally, the comparison of this research work with the reviewed literature is provided. The datasets generated in this research work can be consulted in the Supplementary Materials.

### 4.1. Visual Exploration

**Figure 3:**
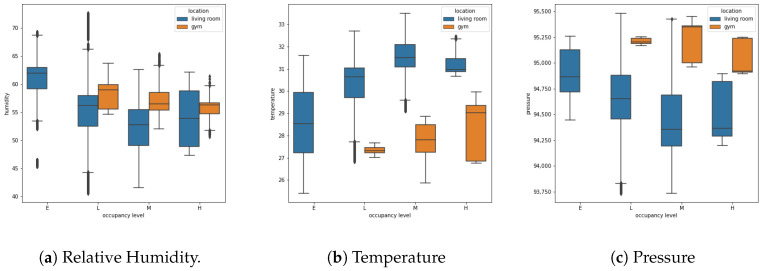
Data distribution for (**a**) relative humidity, (**b**) temperature, and (**c**) pressure grouped by occupancy level and location. Where the occupancy levels are E = Empty, L = Low, M = Medium, and H = High.

Figure 3 shows the distribution of humidity, temperature, and pressure grouped by location. In the case of the fitness gym, there is a tendency for humidity to decrease as occupation increases. For the case of temperature, similar behavior is observed but in the opposite direction, that is, the temperature tends to increase when the occupancy level also increases. Regarding pressure, the values are more dispersed, therefore, there is no clear relationship with respect to the occupation. On the other hand, in the case of the living room, the data behave in a similar way to the data collected in the fitness gym; however, lower average values are observed for humidity and higher average values for temperature. In the case of pressure, contrary to the fitness gym, a trend is observed in relation to occupation, i.e., as the occupancy increases on average, the pressure value decreases.

**Figure 4:**
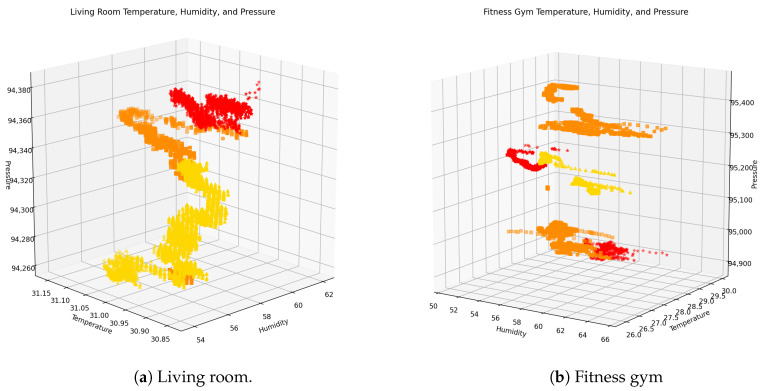
3D scatter plot based on the (**a**) living room and (**b**) fitness gym data. The occupancy levels are indicated as follows: low (yellow), medium (orange), and high (red).

Figure 4 shows the living room and fitness gym data in three axes: humidity, temperature, and pressure. The data of the living room correspond to a single day of the collection while the entire set of data is shown for the case of the fitness gym. The occupancy level is indicated using the following color code: yellow for low occupancy, orange for medium occupancy, and red for high occupancy.

The most noticeable observation is the existence of a clear pattern of data separation in both cases. For the living room, the data with high occupancy are grouped at the top of the graph while the data with low occupancy is grouped at the bottom, leaving most of the data with medium occupancy in the center. No data of empty occupation were registered on this observed day. On the other hand, in the case of the fitness gym, it is observed that the data with medium and high occupancy are grouped at the top and bottom of the graph while the data with low occupancy are closer to the center. The observed differences between these two locations suggest that occupancy estimation is constrained by the characteristics of each space, making it difficult to establish a generalized model. That is, an occupancy prediction model trained with data from one enclosed space will hardly perform well when tested in another enclosed space that does not embrace similar characteristics. Further experimentation must be carried out regarding this matter.

**Figure 5:**
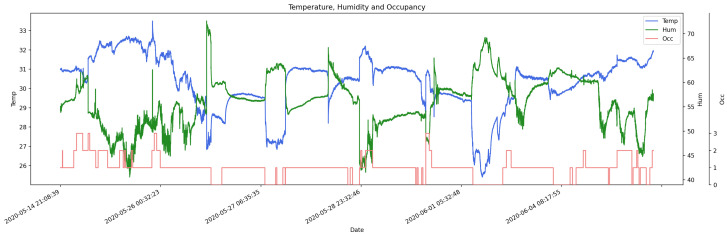
Humidity (green), temperature (blue), and occupancy (red) timeline of the living room data. Time gaps are not shown and occupancy is encoded from zero (Empty) to three (High).

In previous works, it was established that for enclosed places the temperature and humidity values show the opposite behavior when people are not in situ. While when humans are present, this pattern is disturbed in a specific way, which differs from disturbances caused by other factors [5,10,12]. Therefore, such behavior is key to identify the occupation. Figure 5 shows the humidity, temperature, and occupancy timeline for the living room dataset. The gaps between the collection sessions were removed to facilitate the reading of the graph. It is important to highlight that in the periods of empty occupation, the opposite behavior between humidity and temperature is observed, specifically, a lower temperature with a higher relative humidity is presented, compared with occupied periods.

### 4.2. Experimentation Setup

All the experiments were carried out in a laptop computer with the following specifications: 2.2 GHz 6-Core Intel i7 processor and 16 GB 2400 MHz DDR4 of RAM. In each experiment, an exhaustive search was performed on the training set to find the optimal parameters for every algorithm. Table 3 summarizes the optimal parameters found in each case. Regarding the Machine Learning algorithms, the Python programming language, and the Scikit Learn library [30] were used. The Scikit Learn library provides open-source implementations for the three selected algorithms (DT, kNN, and SVM).

### 4.3. Fitness Gym

As mentioned in Section 3.7, for the feature selection the averaged 10 s dataset was used (which contains nine attributes) to generate four datasets with different subsets of features. These datasets were named: FULL, RFE, KBEST, and MIN. The subset of features that each dataset contains is specified in Table 4, with FULL being the dataset with all the features and MIN being the dataset with only the humidity, temperature, and pressure features. In the case of the KBEST and RFE datasets, the five best features were selected using the technique indicated in the name of the dataset. Table 5 shows the results of the experimentation. In general, all the datasets reached a high averaged accuracy (at least 0.96), however, the one that obtained the best score was MIN with an averaged accuracy of 1.0. On the other hand, the FULL dataset achieved the lowest score (0.966). The RFE and KBEST datasets obtained scores very close to 1.0, positioning them between the FULL and MIN datasets. Regarding the algorithms, it can be noticed that the difference in the averaged accuracy between DT (highest) and kNN (lowest) is 0.011, no significant difference is noted.

Regarding the resolution selection, datasets were generated with the following resolutions: 10 s, 30 s, and 1 min. An attempt was made to use a resolution of 5 min, but due to the dataset was too small this idea was discarded. Subsequently, for each resolution, two datasets were generated: the first takes a single sample per interval, and the second averages the corresponding humidity, temperature, and pressure values that lie within the interval. Hence, a total of six datasets were generated by using the subset that obtained the best performance evaluation: the MIN dataset. These datasets were used to perform the occupancy prediction, concentrating in Table 6 the accuracy scores obtained. The best-evaluated datasets were the 30-s datasets with 1 sample and the 1-min averaged datasets, both with an averaged accuracy of 1.0. Regarding the algorithms, kNN averaged and kNN with 1 sample are the best evaluated with an averaged accuracy of 1.0 and 0.998, respectively.

### 4.4. Living Room

Feature selection was performed similarly to the process described for the fitness gym. The features corresponding to each subset are shown in Table 7. It should be noticed that the RFE and KBEST datasets only differ in one feature to their corresponding pairs in the previous experiment, i.e., both methods swapped humidity kurtosis by temperature kurtosis. Hence, the difference between both subsets is just one attribute. Table 5 shows the results of the experiments. The MIN and FULL datasets were again the subsets of features with the highest and lowest performance with an accuracy of 0.976 and 0.866, respectively. Presenting a difference of 0.11, which is three times more than the difference presented in the previous experiment (0.034). In this experiment, the DT algorithm achieved the best-averaged accuracy of 0.956 followed by the kNN algorithm with an averaged accuracy of 0.903. Nevertheless, the SVM results are incomplete due to excessive execution time. By comparing the results of this experiment with the fitness gym is evident that the MIN dataset is the best subset of features and DT had the best-averaged accuracy. However, DT cannot be declared as the best algorithm as only one resolution was used for the experimentation. Therefore, it is necessary to first review the results of the resolution selection experimentation to make a decision.

For the resolution selection, the datasets were generated following the same procedure described for the fitness gym. Additionally, for this experiment, it was possible to generate a dataset with a resolution of five minutes; thus, a total of eight datasets were generated. Table 8 shows the results of experimentation. Overall, the best resolutions are 10 s averaged and 10 s with 0.982 and 0.98, respectively. The difference between the best and worst resolution is 0.1. However, by not taking into account the five minutes datasets this difference is reduced to 0.03 roughly. This suggests that a five minutes resolution loses too much information and impacts negatively on the performance of the models. Comparing these results with the fitness gym results, an important difference is identified: on the fitness gym results, there is no clear trend that the prediction improves with an increment in resolution. This could be due to the difference in the amount of data between the two datasets (the living room datasets have approximately 30 times more data than its fitness gym counterpart), but it could also be due to the difference in the occupancy patterns described in Section 4.1. Hence, further research is required to clearly explain this matter.

Regarding the algorithms, kNN with the averaged datasets has the best score with an accuracy of 0.968 roughly. Furthermore, if the comparison is limited to datasets of the same type (averaged vs. 1 sample), kNN has the best-averaged accuracy in both cases. On the other hand, DT is the worst algorithm with an accuracy of 0.933, and the difference between the best and the worst models is 0.033. Comparing these results with the fitness gym results, a congruence between both results is observed; hence, kNN is considered as the best algorithm overall.

Another result to highlight is that all the averaged scores outperformed their 1-sample counterparts, which suggests that, in general, the averaged datasets are more suited to predict occupancy levels. This conclusion seems reasonable since the averaged datasets have more information summarized. However, shall not pass unnoticed that this extra information comes with a trade-off in the computational resources required to collect, store, and process that information. This also applies to the high-resolution datasets; for example, the number of training instances corresponding to the 10 s and 30 s datasets are approximately 23 k and 2 k, respectively. Furthermore, since the difference between the averaged accuracy of both resolutions is just 0.012, it is clear that the strengths and weaknesses must be carefully analyzed.

### 4.5. Comparison with Other Approaches

From the related work, the six works most similar to the approach proposed in this article were selected to perform a comparison. These works were based mainly on environmental variables to estimate the level of occupation. Table 9 shows the accuracy obtained in each case. The last two columns correspond to the results obtained in this research. It is evident that none of the related works comes close to the results obtained in this research. The closest of them is Adeogun et al. [12] with an accuracy of 0.91; however, they used ten variables and a PIR sensor. It should be noted that all the remaining works used CO${}_{2}$ either individually or together with other variables to predict occupation. Nevertheless, none of them reached an accuracy equal to or greater than 0.90. Another aspect in favor of the approach proposed in this work is that the variables used can be collected using a single sensor, which applies only to Jiang et al. [22] and Zhou et al. [11]; however, they have a significantly lower score than the one obtained in this work.

Viani et al. [8] is the work closest to the approach proposed here. Although they used the humidity and temperature as environmental variables, and they also focused on occupancy prediction (OR = 2), the accuracy that they obtained was lower than in this work, with 0.82 and 0.98, respectively. Nevertheless, it is important to notice that the experiments’ scale was significantly different between both works, with Viani et al. [8] using 29 sensor nodes in a much larger space (a whole museum). Therefore, this suggests that analyzing the relation between scale and accuracy is of utter importance.

Regarding the environmental variables, another variable relevant to the occupancy field is Volatile Organic Compounds (VOCs). VOC measurement has proven to be a viable approach capable of achieving a high accuracy, comparable to that obtained with CO${}_{2}$ [31]. However, Pedersen et al. [32] found a significant drawback that must be taken into account: after first arriving at the monitored space, detection by VOC sensors was delayed more than three-fold compared to CO${}_{2}$ or relative humidity (39 min vs. 12 min) detection. The authors strongly recommended the use of VOC sensors in conjunction with other sensors with quicker detection times in order to obtain a high accuracy when estimating occupancy. Furthermore, the cost of VOC gas sensors can be as much as four times the cost of humidity/temperature sensors. For these reasons, an approach using temperature, humidity, and barometric pressure is considered more convenient for a low-cost solution.

Another alternative approach to detect occupancy is through statistical analysis. Zemouri et al. [33] compared the performance of Machine Learning to detect occupancy against an algorithm developed using statistical analysis, obtaining accuracies of 0.82 and 0.87, respectively. Although they obtained a higher accuracy using statistical analysis, the authors highlighted two significant drawbacks of this approach: (1) the requirement of expert knowledge in the subject area and knowing the physical properties of the variables measured; (2) the slower and more costly performance of this approach, compared to Machine Learning, due to the manual extraction of features. For these reasons, a Machine Learning approach was preferred over statistical analysis in this work. From the literature review, in Section 2 it can also be observed that Machine Learning techniques are the most accepted approach because of the encouraging results obtained with them.

Regarding the occupancy information, this work focused on estimating the occupancy level instead of estimating the exact number of people. According to Jiang et al. [22], two significant benefits can be drawn from this approach: (1) in a dense occupancy environment, fewer low-cost sensors would be necessary for obtaining a high accuracy using occupancy levels rather than the exact number of occupants. (2) For many demand-driven control systems, like Central Domestic Hot Water and building management systems, occupancy levels are adequate to achieve the most energy-efficient control strategy. Hence, occupancy estimation by level is a viable approach that allows the occupancy information systems to be scaled at an efficient cost.

Additionally, the datasets generated in this investigation are considered an important contribution, since no public dataset could be found that offered information on the number of occupants or the level of occupation. Therefore, it is considered that there is a gap in the field of occupation research. In Section 2, it was mentioned that two reliable datasets were found. The first one is the UCI dataset [18], which is an important reference because it is well documented and has been used in multiple works before. However, this dataset offers only binary information regarding the occupation, i.e., if the space is occupied or empty. The other one is the Ecobee’s Donate Your Data program [19], which completely lacks the occupancy ground truth. Therefore, these datasets do not fill the aforementioned gap, nor could it be used for comparison with this work.

It is also important to stress this work has internal and external sources of uncertainty that may have influenced the observed results. First, as it was mentioned in Section 3.3, the subjectivity of the observed occupancy in the fitness gym may be considered a source of uncertainty, with a negative or positive impact on the results. Second, different occupancy levels were used between locations. In the fitness gym, three levels (high, medium, and low) were used, while in the living room an additional empty level was used to complete a total of four levels. Since information related to the empty level was not obtained from the fitness gym, it was not possible to measure the direct impact of the number of levels on the model’s accuracy. Third, external weather conditions are another factor that may influence the results. As mentioned in Section 1, this work does not directly address the impact of weather on the building’s internal environment conditions. However, it is important to stress the collections were carried out in four different months over two years. Those months and their respective average temperatures are September (26.2 ${}^{{}^{\circ}}$C) and October (22.4 ${}^{{}^{\circ}}$C) for the fitness gym, and May (26.2 ${}^{{}^{\circ}}$C) and June (27.9 ${}^{{}^{\circ}}$C) for the living room [34]. These months can be considered warm rather than cold, with the exception of October, which has an average temperature of at least 4 ${}^{{}^{\circ}}$C lower than the others. Even though differences were described in ground truth collection, number of levels, and outside weather, the results from the fitness gym and the living room are congruent in terms of the accuracy obtained for each algorithm in each scenario. This provides confidence that the obtained results are robust and the ground truth collection of the fitness gym had a minimal impact.

## 5. Conclusions and Further Work

In this work, the use of environmental variables (indirect method) and Machine Learning algorithms to estimate occupancy was explored. In the present study, two datasets that concentrate information on environmental variables (humidity, temperature, and pressure) and corresponding occupancy levels were generated from different enclosed spaces. These datasets will allow comparing the effectiveness of the different techniques used by researchers in a standardized way. Thereby, it was possible to estimate the occupancy with an accuracy of at least 97% using the kNN algorithm. This accuracy exceeds previous results found in related works.

It is important to highlight that this accuracy was achieved under the following factors: the use of few environmental variables (i.e., humidity, temperature, and pressure); the exclusion of CO${}_{2}$ in the model because it is difficult to take accurate measurements in real-life environments due to the need to consider all the airflow sources; and the use of well-known Machine Learning models combined with a correct establishment of occupancy levels. In this research work, kNN was selected as the best algorithm to estimate the occupation. However, because of its $O(n^{2})$ time complexity, DT and SVM algorithms might be considered for future developments insomuch as both achieved a similar accuracy (0.95), using the averaged living room dataset.

As future work, exploring the temporal dependence of data on the occupancy level should be considered. Similarly, researching external factors (weather conditions) on the accuracy of estimation should be taken into consideration. In addition, collecting information from other similar enclosed environments and developing an IoT architecture for estimating occupancy using the FIWARE open-source platform are also contemplated.

## Figures and Tables

**Table 1:** Summary of the reviewed related work.

Author	Year	Variables	Algo.	OR	SR	TR	BR	GT	Demo Scale	Dataset
Viani et al. [8]	2014	temp., hum., CO${}_{2}$	SVM	2	3	N/A	0.82 acc.	N/A	29 IoT nodes	own
Cali et al. [20]	2015	CO${}_{2}$ Prof., CO${}_{2}$, windows, doors	Mass balance	2	3	N/A	0.79 custom.	man.	8 d., 5 rooms	own
Fiebig et al. [9]	2017	VOC, Network, Bt. K.F., calendar	MLP, kNN, DT, RF	1	1	N/A	0.75 F1	man.	52 d., 2 bedroom apt.	own
Parise et al. [13]	2019	temp., hum., CO${}_{2}$, PIR	SVM	1	3	10 s	0.96 F1	N/A	2 weeks, 1 room	own
Adeogun et al. [12]	2019	press, hum., CO${}_{2}$, PIR, windows, doors	FNN	2	3	5 min	0.91 acc.	man.	6 weeks, 2 off., 4 pers.	own
Zemouri et al. [10]	2019	temp., hum.	LR, LDA, kNN, CART, NB, SVM, GB	1	3	5 min	0.83 acc.	video	37 d., 1 off., 3 pers.	own
Huchuck et al. [7]	2019	temp., hum., o. temp., PIR, others	LR, MM, HMM, RF, LSTM	1	3	30 min	0.75 acc.	No	1 year, 100 tstat.	DYP [19]
Chitu et al. [21]	2019	CO${}_{2}$, Air Flow	RF, ELM	2	3	1 min	69.17 acc.	algo.	15 d., 4 rooms	USD-OD [23]
Jiang et al. [22]	2020	CO${}_{2}$	Bayessian Filtering with ELM and IMM	2	3	15 min	0.77 acc.	video	30 d., 1 off., 28 pers.	own
Kumar et al. [5]	2020	temp., hum., hum. ratio, CO${}_{2}$, light	ELM	1	3	1 min	0.99 acc.	photos	building office	UCI [18]
Zhou et al. [11]	2020	CO${}_{2}$	gcForest	2	2	1 min	0.82 acc.	video	20 d., 1 lab., 4 pers.	own
Yuan et al. [1]	2020	IR array sensor, temp.	IHMM	2	3	N/A	0.81 acc.	video	6 IoT nodes, 1 lab.	own

**Columns:** Algorithms (Algo.), Occupancy Resolution (OR), Space Resolution (SR), Time Resolution (TR), Best Result (BR), Ground Truth (GT). **Variables:** Temperature (temp.), humidity (hum.), CO2Accumulation (CO2), CO2Occupancy Profile (CO2Prof.), Windows State (windows), Doors State (doors), Network Connections (Network), Bluetooth Key Fobs (Bt. K.F.), Pressure (press), outdoor temperature (o. temp.), infrared(IR), Volatile Organic Compound (VOC). **Algorithms:** SVM (Support Vector Machine), MLP (Multilayer Perceptron), kNN (k-Nearest Neighbor), DT (Decision Tress), RF (Random Forest), FNN (Feedforward Neural Network), LR (Logistic Regression), LDA (Linear Discriminant Analysis), CART (Classification and Regression Trees), NB (Naive Bayes), GB (Gradient Boost), MM (Markov Model), HMM (Hidden Markov Model), LSTM (Long Short-Term Memory), ELM (Extreme Learning Machine), gcForest (Multigrain Cascade Forest), IMM (Inhomogeneous Markov Model), IHMM (Inhomogeneous Hidden Markov Model). **Occupancy Resolution:** (1) Detection, (2) Estimation. **Space Resolution:** (1) Building, (2) Floor, (3) Room. **Best Result:** ACC (Accuracy), F1 (F1 Score), Custom (in a period, percentage of time the algorithm was correct). **Ground Truth:** M (manual), V (video), P (photograph). **Demo Scale:** D (days), W (weeks), Y (years), off. (office), apt. (apartment), lab. (laboratory), pers. (person), tstat (thermostat).

**Table 2:** Datasets before and after being balanced.

Dataset	Before				After			
**Class**	**Empty**	**Low**	**Medium**	**High**	**Empty**	**Low**	**Medium**	**High**
Living room 10 s	4098	16,325	2830	547	16,353	16,325	16,330	16,329
Living room 30 s	1345	5450	956	188	5445	5450	5436	5452
Living room 1 min	673	2730	480	92	2720	2730	2720	2732
Living room 5 min	136	553	96	19	556	553	555	554
Gym 10 s	N/A	202	430	189	N/A	430	430	430
Gym 30 s	N/A	149	149	59	N/A	149	149	150
Gym 1 min	N/A	33	77	34	N/A	77	77	77
Gym 5 min	N/A	21	9	5	N/A	21	21	21

**Table 3:** Parameters used for Features Selection and Resolution Selection experimentation.

Location	Dataset	DT		kNN		SVM	
		Criterion	Max Depth	Algorithm	Neighbors	c	Gamma
Feature Selection Fitness Gym	FULL	entropy	10	ball_tree	1	10	1
	RFE	entropy	6	ball_tree	1	1	1
	KBEST	entropy	10	ball_tree	1	1	1
	MIN	entropy	8	ball_tree	1	1	1
Feature Selection Living Room	FULL	gini	12	ball_tree	1	N/A	N/A
	RFE	gini	12	ball_tree	1	N/A	N/A
	KBEST	gini	12	ball_tree	1	N/A	N/A
	MIN	gini	12	ball_tree	1	N/A	N/A
Resolution Selection Fitness Gym	10 s	entropy	8	ball_tree	1	1	1
	10 avg.	entropy	10	ball_tree	1	1	1
	30 s	entropy	16	ball_tree	1	1	1
	30 avg.	entropy	14	ball_tree	1	1	1
	1 min	entropy	14	ball_tree	1	1	1
	1 avg.	gini	16	ball_tree	1	1	1
Resolution Selection Living Room	10 s	entropy	22	ball_tree	1	100	10
	10 avg.	entropy	24	ball_tree	1	100	10
	30 s	entropy	22	ball_tree	1	100	10
	30 avg.	entropy	22	ball_tree	1	100	10
	1 min	entropy	20	ball_tree	1	100	10
	1 avg.	gini	20	ball_tree	1	100	10
	5 min	entropy	18	ball_tree	1	100	10
	5 avg.	entropy	12	ball_tree	1	100	10

**Table 4:** Subsets of features for the fitness gym dataset. An X indicates a selected feature.

	Humidity			Temperature			Pressure		
	**Mean**	**Kurt**	**Std**	**Mean**	**Kurt**	**Std**	**Mean**	**Kurt**	**Std**
FULL	X	X	X	X	X	X	X	X	X
RFE	X			X	X	X	X		
KBEST	X			X	X		X	X	
MIN	X			X			X		

**Table 5:** Feature selection results for the living room and the fitness gym. Individual scores are shown in green, and mean scores in yellow. A stronger color indicates a higher score.

	Living Room					Fitness Gym				
	**FULL**	**RFE**	**KBEST**	**MIN**	**Mean**	**FULL**	**RFE**	**KBEST**	**MIN**	**Mean**
SVM	NA	NA	NA	NA		0.9512	1.0	0.9951	1.0	0.9866
kNN	0.7868	0.9231	0.9172	0.9848	0.9030	0.9512	1.0	0.9853	1.0	0.9841
DT	0.9441	0.9562	0.9586	0.9663	0.9563	0.9951	0.9951	0.9902	1.0	0.9951
Mean	0.8655	0.9397	0.9379	0.9756		0.9658	0.9984	0.9902	1.0	

**Table 6:** Resolution selection results for the fitness gym location. Individual scores are shown in green and mean scores in yellow. A stronger color indicates a higher score.

	1 Sample				Averaged			
	**10 s**	**30 s**	**1 min**	**Mean**	**10 s**	**30 s**	**1 min**	**Mean**
SVM	0.9951	1.0	1.0	0.9984	1.0	0.9857	1.0	0.9952
kNN	0.9951	1.0	1.0	0.9984	1.0	1.0	1.0	1.0
DT	1.0	1.0	0.9722	0.9907	0.9902	1.0	1.0	0.9967
Mean	0.9967	1.0	0.9907		0.9967	0.9952	1.0	

**Table 7:** Subsets of features for the living room dataset. An X indicates a selected feature.

	Humidity			Temperature			Pressure		
	**Mean**	**Kurt**	**Std**	**Mean**	**Kurt**	**Std**	**Mean**	**Kurt**	**Std**
FULL	X	X	X	X	X	X	X	X	X
RFE	X	X		X		X	X		
KBEST	X	X		X			X	X	
MIN	X			X			X		

**Table 8:** Resolution selection results for the living room location. Individual scores are shown in green and mean scores in yellow. A stronger color indicates a higher score.

	1 Sample					Averaged				
	**10 s**	**30 s**	**1 min**	**5 min**	**Mean**	**10 s**	**30 s**	**1 min**	**5 min**	**Mean**
SVM	0.9716	0.9652	0.9517	0.9054	0.9485	0.9756	0.9657	0.9657	0.8955	0.9506
kNN	0.9848	0.9753	0.9577	0.9104	0.9571	0.9882	0.9768	0.9607	0.9452	0.9677
DT	0.9825	0.9712	0.9436	0.8358	0.9333	0.9831	0.9743	0.9476	0.9054	0.9526
Mean	0.9796	0.9706	0.9510	0.8839		0.9823	0.9723	0.9580	0.9154	

**Table 9:** Comparison of best results with results in the related works.

	Viani et al. [8]	Adeogun et al. [12]	Chitu et al. [21]	Jiang et al. [22]	Zhou et al. [11]	Fitness Gym	Living Room
Accuracy	0.82	0.91	0.69	0.77	0.82	1.0	0.98
Variables	temp. hum. CO${}_{2}$	temp. pre. hum. 7 more	CO${}_{2}$ air flow	CO${}_{2}$	CO${}_{2}$	temp. hum. pre.	temp. hum pre.

## Supplementary Materials

Data for both scenarios are available at this link: https://github.com/AndreeVela/occupancy_estimation. Files include the original data, as well as the data aggregated at different time resolutions and the data used for feature selection.

## Abbreviations

The following abbreviations are used in this manuscript:

AC	Air Conditioner
ADASYN	Adaptive Synthetic
BLE	Bluetooth Low Energy
CART	Classification and Regression Trees
DT	Decision Trees
EC	Edge Computing
ELM	Extreme Learning Machine
FNN	Feedforward Neural Network
GB	Gradient Boost
HMM	Hidden Markov Model
HVAC	Heating, Ventilation, and Air Conditioners
IEA	International Energy Agency
IoT	Internet of Things
kNN	K-Nearest Neighbor
LDA	Linear Discriminant Analysis
LR	Logistic Regression
LSTM	Long Short-Term Memory
MLP	Multilayer Perceptron
MM	Markov Model
PIR	Passive Infrared
RF	Random Forest
RFE	Recursive Feature Elimination
RFID	Radio Frequency Identification
SRAM	Static Random Access Memory
SVM	Support Vector Machine
UCI	University of California, Irvine
VOC	Volatile Organic Compound
WiFi	Wireless Fidelity
